# Functional Complementation of a Model Target to Study Vpu Sensitivity

**DOI:** 10.1371/journal.pone.0068507

**Published:** 2013-06-28

**Authors:** Sanath Kumar Janaka, Jared Faurot, Marc C. Johnson

**Affiliations:** Department of Molecular Microbiology and Immunology, University of Missouri, Columbia, Missouri, United States of America; Helmholtz Zentrum Muenchen - German Research Center for Environmental Health, Germany

## Abstract

HIV-1 forms infectious particles with Murine Leukemia virus (MLV) Env, but not with the closely related Gibbon ape Leukemia Virus (GaLV) Env. We have determined that the incompatibility between HIV-1 and GaLV Env is primarily caused by the HIV-1 accessory protein Vpu, which prevents GaLV Env from being incorporated into particles. We have characterized the ‘Vpu sensitivity sequence’ in the cytoplasmic tail domain (CTD) of GaLV Env using a chimeric MLV Env with the GaLV Env CTD (MLV/GaLV Env). Vpu sensitivity is dependent on an alpha helix with a positively charged face containing at least one Lysine. In the present study, we utilized functional complementation to address whether all the three helices in the CTD of an Env trimer have to contain the Vpu sensitivity motif for the trimer to be modulated by Vpu. Taking advantage of the functional complementation of the binding defective (D84K) and fusion defective (L493V) MLV and MLV/GaLV Env mutants, we were able to assay the activity of mixed trimers containing both MLV and GaLV CTDs. Mixed trimers containing both MLV and GaLV CTDs were functionally active and remained sensitive to Vpu. However, trimers containing an Env with the GaLV CTD and an Env with no CTD remained functional but were resistant to Vpu. Together these data suggest that the presence of at least one GaLV CTD is sufficient to make an Env trimer sensitive to Vpu, but only if it is part of a trimeric CTD complex.

## Introduction

Unlike most gammaretrovirus Env proteins, GaLV Env cannot pseudotype HIV-1 particles [1], [2]. This incompatibility was found to be dictated by the CTD of GaLV Env [1], [3], [4]. Recently, we and others have reported that this incompatibility is primarily caused by the HIV-1 accessory protein Vpu, which prevents Env proteins with the GaLV Env CTD from being incorporated into viral particles [3], [4], [5]. The mechanism of GaLV Env modulation by Vpu is not completely understood. Vpu reduces the expression of GaLV Env in the cell, but the loss of expression does not correlate with the loss of infectivity. Most notably, infectious particle output is dramatically reduced in the presence of Vpu even when significant GaLV Env remains expressed on the cell surface [4]. A comprehensive mutagenic scan of the cytoplasmic domain of Vpu in its native proviral context revealed a nearly perfect overlap in the Vpu sequences in the cytoplasmic domain required for modulation of GaLV Env and BST-2/Tetherin [6]. However, unlike BST-2/tetherin, the membrane spanning domain of Vpu does not appear to play an important role in GaLV Env modulation [4], [6].

The CTDs of gammaretroviral Env proteins are predicted to be alpha helical and likely form coiled-coil trimers [7], [8], [9]. An alanine scanning mutagenic analysis of the GaLV Env CTD helped identify the sequence conferring Vpu sensitivity on the GaLV Env CTD to be INxxIxxVKxxVxRxK [5]. The motif contains two lysine residues that are also found in the corresponding positions of the CD4 CTD, another target of Vpu [5]. We observed that mutation of the hydrophobic residues believed to form the interface of the CTD coiled-coil alleviated Vpu sensitivity. We therefore wanted to understand if this putative coiled-coil is required for the Env trimer to be Vpu sensitive, and whether all three helices in this trimer must contain the sensitivity motif. To answer these questions, we chose to take advantage of functional complementation of receptor-binding defective or fusion defective MLV Envs with the CTDs containing a Vpu-sensitive or a Vpu-resistant helix [10].

The Friend murine leukemia virus (F-MLV) Env protein is a trimer [11]. F-MLV Env is produced as an 85 kDa precursor in the ER, where it trimerizes. Upon transport of the trimer through the Golgi network, Env maturation takes place with a furin cleavage event and the individual monomers are made up of a 70 kDa surface subunit (SU) and a 15 kDa transmembrane unit (TM)[12], [13], [14]. SU provides the receptor binding function [15], [16], [17], [18], [19] and TM provides the fusion function [20], [21], [22]. Mutations conferring receptor binding defects and fusion defects have been identified, and some pairs of defects on two separate Env molecules can functionally complement each other [10], [22]. In this study, binding defective (BD) and fusion defective (FD) F-MLV Env and F-MLV/GaLV Env Δ8 mutants were used to generate functionally active Env trimers containing both the MLV and GaLV CTDs. We performed infectivity assays with the complementation pairs in the presence and absence of Vpu to elucidate if mixed trimers remain Vpu sensitive.

## Materials and Methods

### Plasmids

**Figure 1:**
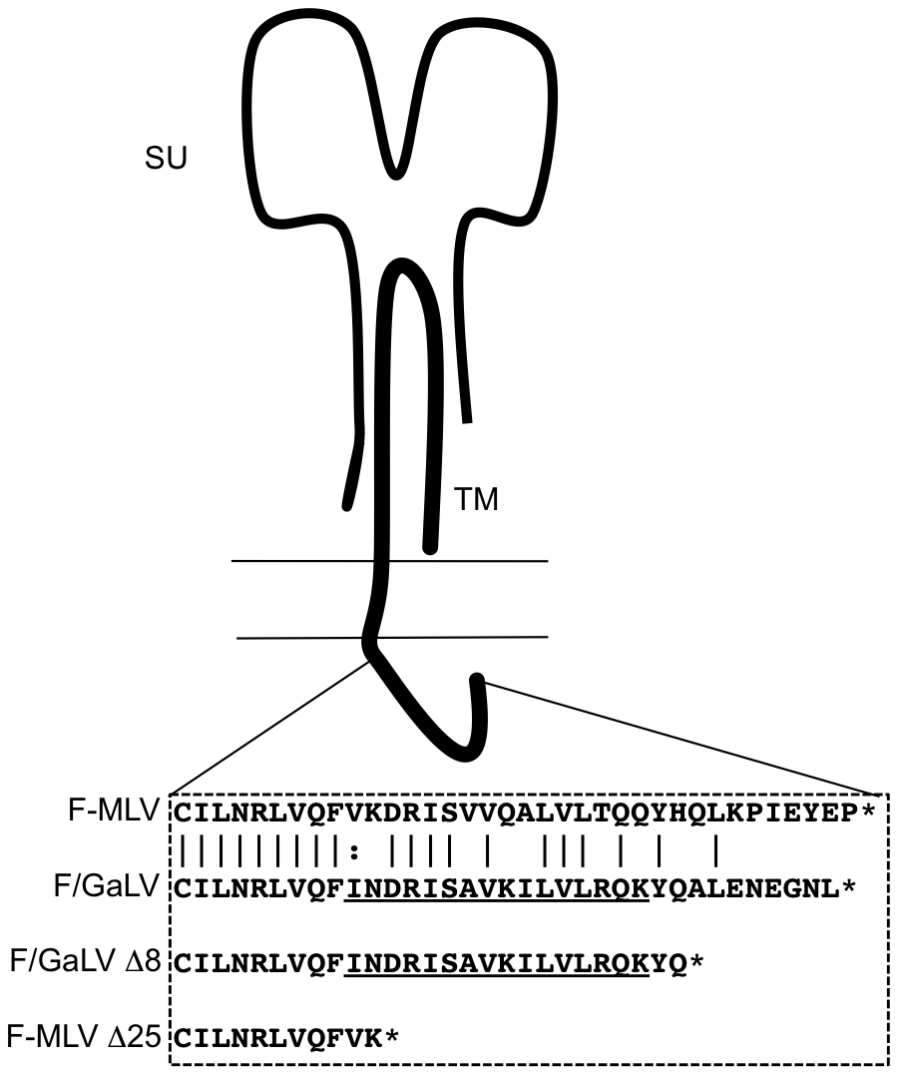
Schematic of the gammaretroviral Env proteins. The amino acid sequences in the CTD of F-MLV Env and the F-MLV/GaLV Env chimera are depicted. Truncations in the CTD of the Envs, F-MLV Δ25 and F-MLV/GaLV Δ8 are indicated. Underlined sequence represents the element that dictates Vpu sensitivity. The cartoon has been adapted from figure 1 of ref. 5.

The ecotropic F-MLV Env (isolate 57) expression construct was kindly provided by Walther Mothes (Yale University). The F-MLV/GaLV Env Δ8 chimeric construct containing an 8- amino acid truncated version of the GaLV Env CTD was constructed using oligonucleotide linkers coding for the cytoplasmic tail of GaLV [5]. These oligonucleotides were inserted between the ClaI site (encoded within DRL amino acid coding sequence, 30 amino acids upstream of the C-terminus), and the EcoRI site that occurs after the stop codon in the cytoplasmic tail region of the F-MLV Env expression construct. The Δ25 mutation in MLV Env was constructed by replacement of the DNA sequence between ClaI and EcoRI with linkers introducing a stop codon after FVK [23] (Fig. 1). Mutations that make the F-MLV Env binding defective (D84K) or fusion defective (L493V) were described earlier and the mutations were introduced into the F-MLV or F-MLV/GaLV construct by Overlap Extension PCR. NL4-3 derived HIV-CMV-GFP was kindly provided by Vineet KewalRamani (National Cancer Institute-Frederick). This vector lacks the genes encoding Vif, Vpr, Vpu, Nef and Env and has a CMV immediate-early promoter driven GFP in the place of Nef. The Vpu^+^ HIV-CMV-GFP was created by replacing the fragment between BamHI and SalI sites in HIV-CMV-GFP with the equivalent BamHI-SalI fragment encoding Vpu from the plasmid ΔR8.2.

### Cell culture

The 293FT cell line was obtained from Invitrogen. The cell line expressing the ecotropic F-MLV Env receptor, 293T mCAT-1[24], was kindly provided by Walther Mothes. The cell lines were maintained in Dulbecco's modified Eagle's medium (DMEM) supplemented with 10% fetal bovine serum, 2 mM glutamine, 1 mM sodium pyruvate and 10 mM non-essential amino acids.

### Infectivity/Vpu sensitivity assays

Infectivity assays using HIV-CMV-GFP and its derivative were performed by transfection of 293FT cells with 500 ng of the HIV proviral DNA and 500 ng total of the Env and 4 µl of 1 mg/ml polyethylenimine (PEI) in six well plates [25]. In the case of the functional complementation of the Env, 250 ng of a binding defective and 250 ng of a fusion defective Env were used. The media were replaced 8 to 12 h post transfection to remove any residual transfection reagent and to allow the cells to recover from the transfection procedure. Supernatant was collected 24 h after the media were exchanged and then frozen at −80°C to lyse any cells in the supernatant. The supernatant was thawed in a 37°C water bath and spun at 1500×g for 10 min to pellet any cells or cell debris. 500 µl virus supernatants were applied to fresh 293T-mCAT1 cells in a 12- well plate. Cells were collected 48 h later, fixed with 4% paraformaldehyde, and analyzed by flow cytometry using Accuri C6 flow cytometer systems. Vpu sensitivity is expressed as a ratio of the percentage of cells infected by HIV-1 lacking Vpu to the percentage of cells infected by HIV-1 containing Vpu. Complementation efficiency is expressed as the ratio of percentage of cells infected with Vpu^-^ HIV-1 pseudotyped with a pair of complemented Envs to the percentage of cells infected with Vpu^-^ HIV-1 pseudotyped with F-MLV Env in the same experiment. Vpu sensitivity for each complementation pair is defined as the ratio of infectivity in the absence of Vpu to the infectivity in the presence of Vpu.

## Results and Discussion

Within the Env CTD, F-MLV and GaLV Env sequences are highly similar (Fig.1). In the presence of Vpu, glycoproteins with a GaLV Env CTD are prevented from being incorporated into HIV-1 particles [3], [4]. To expand on these studies and to understand the physical target of Vpu, as mentioned earlier, we chose to take advantage of Env complementation. Functionally defective Env constructs lacking receptor binding function or fusion function were created, as described previously [10]. The D84K mutation, conferring a receptor binding defect (BD) on the Env and the L493V mutation, conferring a fusion defect (FD) on the Env are able to functionally complement one another [10]. Env constructs with these mutations were generated in the context of F-MLV with a GaLV Env Δ8 CTD (Vpu sensitive helix), an F-MLV Env CTD (Vpu resistant helix), and an F-MLV Env Δ25 CTD (Fig. 1). GaLV Env Δ8 CTD lacks the C-terminal eight amino acids from GaLV Env and was used because the last eight amino acids of GaLV Env reduces infectivity with HIV-1 cores irrespective of Vpu [5]. F-MLV Env Δ25 CTD lacks the majority of its cytoplasmic tail and was chosen because it remains as infectious as wildtype F-MLV Env when pseudotyped with HIV-1 particles despite being tail-less [23].

### Gammaretroviral Envs with different CTDs complement each other

**Figure 2:**
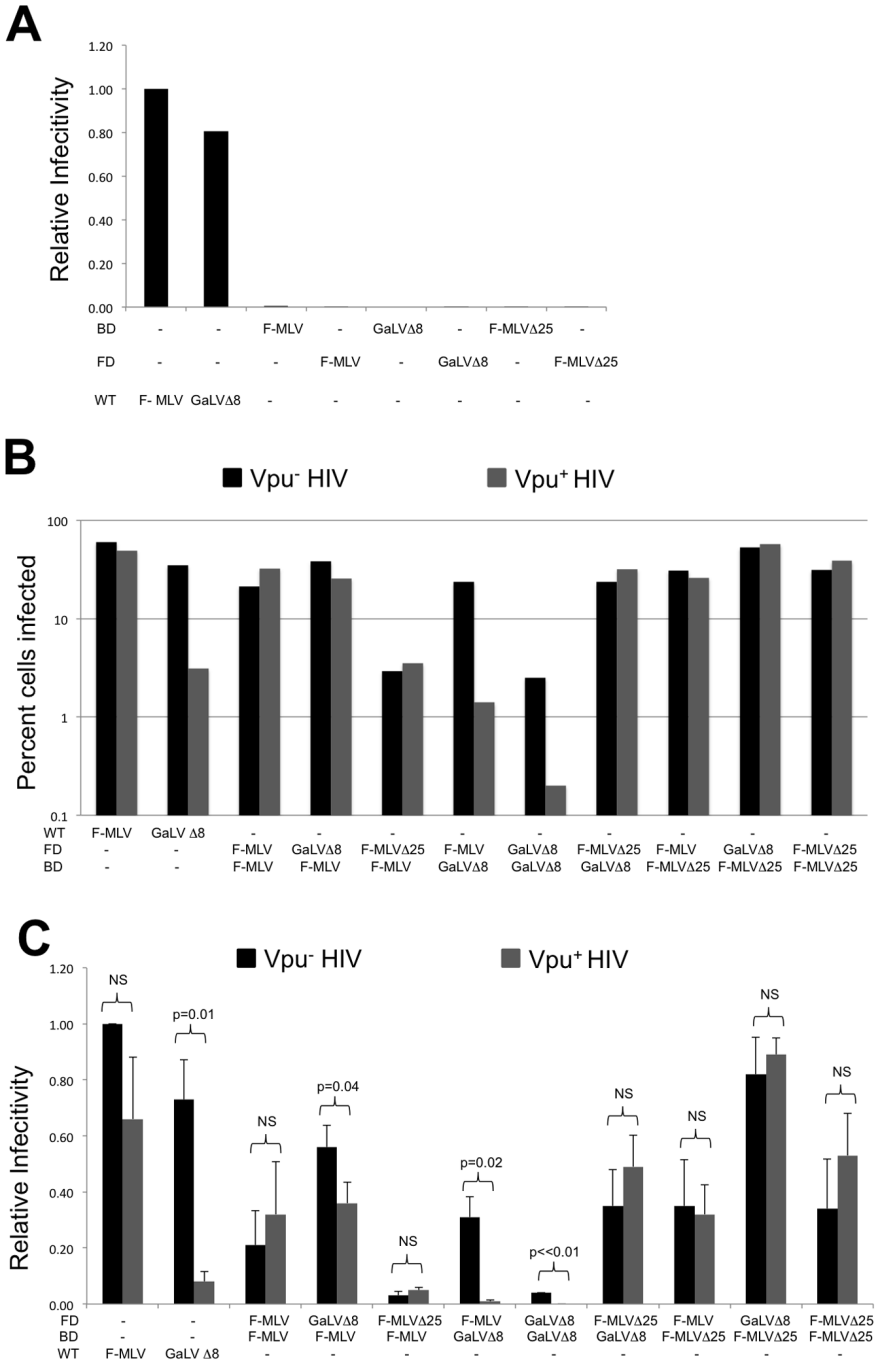
Defective Env pairs with different CTDs complement each other functionally. (A) Infectivity of the indicated Envs relative to infectivity with F-MLV Env. Data shown is the average of two experiments. (B) Infectivity of complementation pairs in the presence or absence of Vpu, relative to infectivity with F-MLV Env. (C) Average of three experiments with the infectivity normalized to that of F-MLV Env. Error bars indicate SD in the experiments. Welch's T- test was performed to determine p- values, indicative of statistical significance. NS- Non significant; BD- Binding defective; FD- Fusion defective.

Infectivity assays with HIV-CMV-GFP were performed with pairs of BD and FD Env constructs to confirm that the proteins are able to complement each other as previously described [10]. The individual BD and FD Envs do not produce infectious particles on their own (Fig. 2A) but each BD construct was able to complement each FD Env with variable efficiency (Fig. 2B,C). Figure 2B shows the raw infectivity from a representative experiment and Figure 2C shows the average and standard deviation of three experiments where infectivity was normalized to that of wildtype (WT) F-MLV Env. While highly related Envs have been known to form mixed trimers [26], infectivity data from this assay shows that Envs with different CTDs can still complement each other functionally.

### Mixed Env trimers can be Vpu sensitive

**Figure 3:**
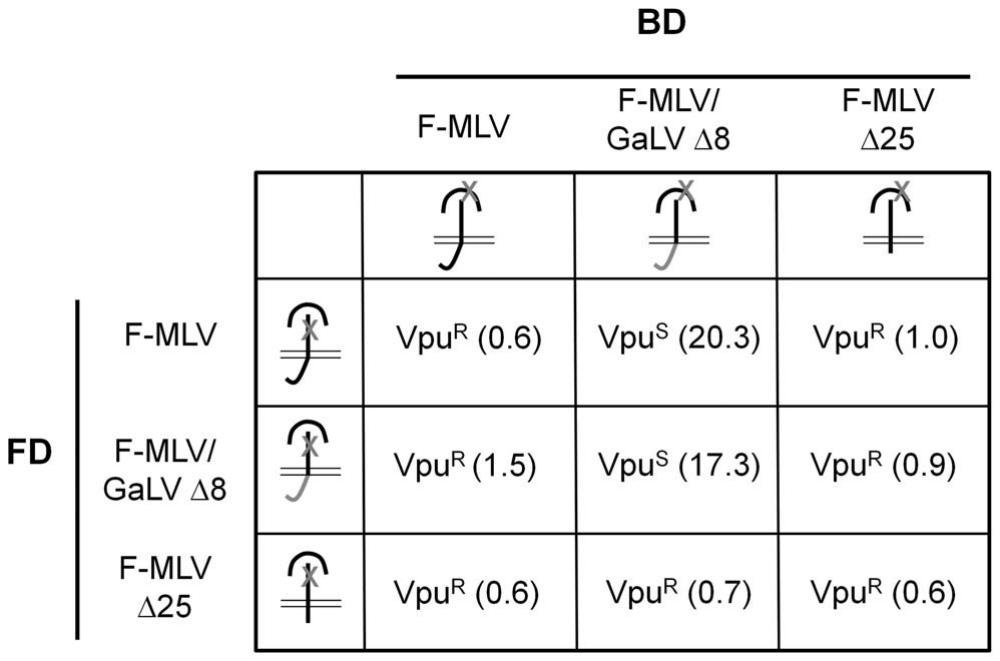
Mixed Env trimers can be Vpu sensitive. Sensitivity to Vpu of each complementation pair is indicated. The degree of Vpu sensitivity is indicated within parentheses as a ratio of infectivity in the absence of Vpu to infectivity in the presence of Vpu. Data shown is the average of three independent experiments in Fig 2B.Vpu^R^- Vpu resistant; Vpu^S^- Vpu sensitive.

To understand the Vpu sensitivity of the different Env trimers, pairwise transfections with complementation pairs were performed in the presence or absence of Vpu as previously described ([5], Fig. 2B,C). Vpu sensitivity was calculated as the ratio of infectivity in the absence of Vpu to the infectivity in the presence of Vpu (Fig. 3). As expected, the Env trimers that contained only F-MLV CTDs were Vpu resistant and the Env trimers that contained only GaLV Env Δ8 CTDs were Vpu sensitive (Fig. 2, 3). With mixed trimers, infectivity of the complementation pair of a BD F-MLV/GaLV Env Δ8 (Vpu sensitive) with an FD F-MLV Env (Vpu resistant) was 20-fold higher in the absence of Vpu (Fig. 2 and 3), suggesting that Vpu sensitivity does not require all three CTDs to contain the sensitivity sequence. However, infectivity of the reciprocal complementation pair, with an FD F-MLV/GaLV Env Δ8 and a BD F-MLV Env, was only 1.5 fold higher in the absence of Vpu (Fig. 2B, 3). Previous complementation studies have demonstrated that maximum complementation requires the expression of excess glycoprotein with a functional fusion domain [10]. Because each glycoprotein contains three units, the functional glycoprotein complementation pairings presumably contain two functional fusion domains and one functional receptor-binding domain. Therefore, the functional timers from the FD F-MLV/GaLV Env Δ8:BD F-MLV Env complementation pair would contain one Vpu sensitive CTD and two Vpu resistant CTDs. The observations that this complementation pair is less sensitive than the reverse pair where the functional timers would contain two Vpu sensitive CTDs (FD MLV Env:BD F-MLV/GaLV Env Δ8) suggests that maximum Vpu sensitivity requires at least two Vpu sensitive helices. Alternatively, the FD mutation could cause an alteration TM's tertiary structure that perturbs Vpu sensitivity, or the SU domain of Env may be able to partially complement independent of its Vpu-sensitive TM subunit. Modularity of the receptor binding function has been previously reported [27].

### Vpu sensitivity is conferred on a functionally complemented Env complex only if all the three CTDs are present

The hydrophobic amino acids in the ‘vpu-sensitivity sequence’ may form a coiled-coil interface in the predicted alpha helix [7], and so we tested whether F-MLV/GaLV Env proteins with fewer than three CTDs could remain Vpu sensitive. For this, we complemented F-MLV Env Δ25 with F-MLV and F-MLV/GaLV Env Δ8. F-MLV Env Δ25 remains functional but lacks the entire Vpu sensitivity element [5], [23]. Despite the lack of CTD, functional complementation occurred with each of these pairs, but the pairs were all Vpu resistant (Fig. 2 and 3). Loving et al. [8] have shown with electron cryotomography reconstructions that the TM of a full length Env is in the form of a closely packed density perpendicular to the membrane prior to R-peptide cleavage. They suggest that TM is held in this closely packed form by CTD-CTD interactions that are relieved by R-peptide cleavage. Together, these pieces of data support a model wherein the gammaretroviral Env CTD forms a trimeric helix bundle and that Vpu sensitivity can only be conferred in the context of this structure.
